# Nanoplastics increase *in vitro* oestrogenic activity of neurotherapeutic drugs

**DOI:** 10.2478/aiht-2024-75-3818

**Published:** 2024-03-29

**Authors:** Lucija Božičević, Valerije Vrček, Nikolina Peranić, Nikolina Kalčec, Ivana Vinković Vrček

**Affiliations:** Institute for Medical Research and Occupational Health, Zagreb, Croatia; University of Zagreb Faculty of Pharmacy and Biochemistry, Zagreb, Croatia; University of Rijeka Faculty of Medicine, Rijeka, Croatia

**Keywords:** polystyrene, carbamazepine, fluoxetine, oestrogen receptors, T47D-KBluc cell line, estrogenski receptori, fluoksetin, karbamazepin, polistiren, stanična linija T47D-KBluc

## Abstract

Environmental pollution with plastic nanoparticles (PNPs) has rendered hazard assessment of unintentional human exposure to neurotherapeutic drugs through contaminated water and food ever more complicated. Due to their small size, PNPs can easily enter different cell types and cross different biological barriers, while their high surface-to-volume ratio enables higher adsorption of chemicals. This is how PNPs take the role of a Trojan horse as they enhance bioaccumulation of many different pollutants. One of the health concerns related to water pollution with neurotherapeutic drugs is endocrine disruption, already evidenced for the anticonvulsant drug carbamazepine (Cbz) and antidepressant fluoxetine (Flx). Our study aimed to evaluate endocrine disrupting effects of Cbz and Flx in mixtures with polystyrene nanoparticles (PSNPs) using the *in vitro* luciferase assay to measure oestrogen receptor activity in T47D-KBluc cells treated with Cbz-PSNPs or Flx-PSNPs mixtures and compare it with the activities observed in cells treated with individual mixture components (Cbz, Flx, or PSNPs). Dose ranges used in the study were 0.1–10 mg/L, 1–100 µmol/L, and 0.1–10 µmol/L for PSNPs, Cbz, and Flx, respectively. Our findings show that none of the individual components activate oestrogen receptors, while the mixtures induce oestrogen receptor activity starting with 0.1 mg/L for PSNPs, 10 µmol/L for Cbz, and 0.5 µmol/L for Flx. This is the first study to evidence that PSNPs increase oestrogen receptor activity induced by neurotherapeutic drugs at their environmentally relevant concentrations and calls for urgent inclusion of complex mixtures in health hazard assessments to inform regulatory response.

## Abstract (hr)

Onečišćenje okoliša nanočesticama plastike (PNP) dodatno je otežalo procjenu opasnosti od nenamjernog izlaganja ljudi neuroterapijskim lijekovima putem kontaminirane vode i hrane. Zbog svoje male veličine, PNP-i mogu lako ući u različite tipove stanica i prijeći različite biološke barijere, a njihov omjer površine i volumena omogućuje adsorpciju veće količine tvari na njihovu površinu. Upravo na taj način PNP-i preuzimaju ulogu trojanskoga konja te pospješuje bioakumulaciju mnogih zagađivača u ljudskom i drugim organizmima. Među štetnim učincima povezanima sa zagađenjem vode neuroterapijskim lijekovima jesu i poremećaji rada endokrinoga sustava. Takvi su učinci već dokazani za antikonvulzivni lijek karbamazepin (Cbz) i za antidepresiv fluoksetin (Flx). Našem je istraživanju cilj bio procijeniti endokrino disruptivne učinke Cbz i Flx u složenim smjesama s nanočesticama polistirena (PSNP) korištenjem luciferaznog eseja za ispitivanje aktivacije estrogenskih receptora u T47D-KBluc stanicama tretiranima smjesama Cbz-PSNP ili Flx-PSNP i usporediti ih s učincima koji su uočeni u stanicama tretiranima pojedinačnim komponentama smjese (Cbz, Flx ili PSNP). U istraživanju su korišteni ovi rasponi doza: 0,1 – 10 mg/L, 1 – 100 µmol/L i 0,1 – 10 µmol/L za PSNP, Cbz i Flx. Rezultati su pokazali kako niti jedna od pojedinačnih komponenti ne aktivira ER, a smjese induciraju aktivnost estrogenskih receptora, počevši od 0,1 mg/L za PSNP, 10 µmol/L za Cbz i 0,5 µmol/L za Flx. Ovo je prva studija koja dokazuje da PSNP-i povećavaju aktivnost estrogenskih receptora izazvanu neuroterapijskim lijekovima u njihovim ekološki relevantnim koncentracijama i poziva na hitno uključivanje procjene opasnosti od izloženosti složenim smjesama u procjene rizika za ljudsko zdravlje.

Endocrine disrupting chemicals (EDCs) raise great health concerns for the global population. The strategic approach to deal with these substances in the European Union has been outlined in the Communication Towards a Comprehensive EU Framework on Endocrine Disruptors (1). EDCs interfere with the endocrine system and adversely affect the development, reproduction, metabolism, and the nervous and immune system (2) as they mimic body hormones that trigger and bind to cell receptors, blocking their interaction with natural hormones. Some EDCs interact with multiple receptors, and multiple EDCs interact with the same receptor (3). Interactions of EDCs with human organism can lead to several disorders such as obesity, diabetes, infertility, and endocrinopathies, as well as to hormone-dependent cancers (4).

Screening and testing for EDCs was initiated as high-priority in 1998 by the Organisation for Economic Co-operation and Development (OECD), which developed the test No. 455 (5) to evaluate oestrogenic activity and EDC interference with normal oestrogen signalling mediated by oestrogen receptors.

EDCs are highly diverse and include phytoestrogens in food, synthetic endocrine-acting chemicals in regulated birth control, hormone replacement and steroid medicines, non-hormonal medicines (such as antipsychotics, antiepileptics, antihypertensives, antivirals, antidiabetics, and anticancer drugs), as well as environmental contaminants (such as pesticides, dioxins, perchlorates, phthalates, and polybrominated diphenyl ethers) (6). Our environment is being polluted with pharmaceuticals of all categories and their metabolites throughout their life cycle, most notably the aquatic environment, in which they are detected in low concentrations (measured in pg/L to μg/L) (7, 8). The general population is mostly exposed to them through drinking water, residues in leaf crops, root crops, fishery products, dairy products, and meat.

Health risks posed by pharmaceuticals in different environmental compartments cannot be clearly distinguished from the total risk of combined exposure with other chemicals and materials with potentially stronger impact on human health than exposure to individual components alone, even at concentrations regarded as safe. In this respect, the European Food and Safety Authority (EFSA) and the OECD have already provided guidance on how to assess risk from such mixtures (9,10,11).

Assessment is additionally challenged by plastic along its value chain. Plastic pollution is now considered one of the greatest environmental issues due to its abundance and persistence in the aquatic environment (12). Plastics undergo various types of mechanical and biological degradation to micro- and nanoparticles (13), and nanoplastics can adsorb and accumulate toxic chemicals from the environment, acting as a “Trojan horse” for hazardous substances (14).

Our intent with this study was to contribute to human health hazard assessment by investigating for the first time the oestrogenic activity of complex mixtures of nanoplastics and neuroactive drugs. To this end we used selected, well characterised, commercially available polystyrene nanoparticles (PSNPs) combined with either carbamazepine (Cbz) or fluoxetine (Flx).

Cbz is an anticonvulsant for the treatment of epilepsy, bipolar disorder, and, more recently, neuropathic pain such as trigeminal neuralgia (15). It is often found in water bodies in a wide range from ng/L to µg/L and average concentration of 11.6 µg/L globally (16) or 12 µg/L in Europe (17). It is mostly neurotoxic as it lowers neuronal excitability, changes transmembrane transport, and regulates several neurotransmitters, but some studies evidence endocrine disruption, which causes imbalance in sex hormones and reproductive function impairment (15, 18, 19).

Flx is the third most prescribed selective serotonin reuptake inhibitor (SSRI) indicated for depression (20). Its average concentrations can reach 1.4 µg/L in the environment (11) and range between 0.5 and 0.8 ng/L in drinking water (21). In surface waters, it can reach 50 ng/L, as reported in the United Kingdom and some parts of Europe (22). Flx can have behavioural, neurotoxic, and endocrine disruptive effects (20, 23, 24). Several *in vitro* studies also reported disruption of steroid hormone production (25, 26) and interaction with oestrogen receptors (27, 28).

## MATERIALS AND METHODS

All testing was done in accordance with the EDC screening programmes devised by the OECD (5) and the United States Environmental Protection Agency (US EPA) (29) using the human-derived cell line T47D-KBluc.

### Characterisation of nanoparticles

PSNPs were purchased as a commercial stock of 25 nm particles in the concentration of 10,500 mg/L from Phosphorex (Hopkinton, MA, USA). They were visualised and their primary size (*d*, nm) determined with a transmission electron microscope (TEM) (JEOL JEM 1010, JEOL, Tokyo, Japan) in samples prepared by suspending them in the cell culture RPMI-1640 medium without phenol red (Sigma Aldrich, Steinheim, Germany) supplemented with 5 % charcoal-stripped foetal bovine serum (CS-FBS) (Sigma Aldrich) to obtain the concentration of 1 mg/L. The suspension was dropped on a Formvar^®^-coated copper grid (SPI Supplies, West Chester, PA, USA), dried overnight at room temperature, and measured in a bright field mode at an acceleration voltage of 80 kV. Images were taken with a Canon PowerShot S50 Camera (Canon, Tokyo, Japan). Results are presented as mean values (*d*, in nm) of 60 particles with standard deviations (SD) calculated with the ImageJ software (LOCI, University of Wisconsin, Madison, WI, USA). Hydrodynamic diameter (*d_H_*) and size distribution were determined for PSNPs suspended in cell culture for 24 h and those not suspended in cell culture with dynamic light scattering and zeta potential using a Zetasizer Ultra instrument (Malvern Panalytical, Malvern, UK). Data were processed with the ZS Xplorer 3.21 software (Malvern Panalytical). The *d*_H_ results represent the means of six measurements expressed as intensity-weighed size distribution (in nm), while the zeta potential represents the means of three ELS measurements expressed in mV.

### Cell culturing

The T47D-KBluc cell line was purchased from the American Type Culture Collection (ATCC, Manassas, VA, USA). These are reporter-labelled cells transfected with triplet oestrogen-responsive elements used to screen for oestrogenic or anti-oestrogenic activity of chemicals (30). T47D-KBluc cells were cultured in tissue culture flasks (Sarstedt, Nümbrecht, Germany) with the RPMI-1640 medium supplemented with 10 % (v/v) CS-FBS and 1 % (v/v) antibiotic-antimycotic solution (Sigma Aldrich) at 37 °C and 5 % CO_2_ to reach the density of 1×10^6^ cells/mL (90–95 % confluence) before treatment.

### Cytotoxicity evaluation

Before we determined oestrogen receptor activity, we established dose-response cytotoxicity for PSNPs, Cbz, and Flx alone and for their mixtures. For that purpose, T47D-KBluc cells were seeded in 12-well plates (Sarstedt, Nümbrecht, Germany) containing 1 mL of cell culture medium at a density of 1×10^5^ cells/well at 37 °C and 5 % CO_2_. After 24 h, we replaced the medium and treated the cells with different concentrations of test substances or their mixtures.

Dose ranges were selected based on literature data on *in vitro* toxicity of PSNPs, Cbz, and Flx (31,32,33) as follows: 0.1–10 mg/L for PSNPs, 3.75–500 µmol/L for Cbz, and 0.75–100 µmol/L for Flx. Their cytotoxicity was first tested with the MTS assay as described elsewhere (34) with the aim to find the doses at which >90 % cells remained viable. Negative controls were untreated cells and positive controls were cells treated with 10 % (v/v) DMSO (Sigma Aldrich).

After 48 h of treatment at 37 °C and 5 % CO_2_, the medium was removed from the plates into 2 mL Eppendorf tubes (Eppendorf, Hamburg, Germany). Cells remaining in the wells were washed with phosphate buffered saline (PBS) three times and then detached by incubating them with Trypsin-EDTA solution (Sigma Aldrich, Steinheim, Germany) at 37 °C and 5 % CO_2_ for 5–7 min. The detached cells were then added to the Eppendorf tubes with previously collected medium.

Due to possible interferences with the MTS assay, cytotoxicity testing was then repeated using flow cytometry for PSNP doses of 0.1, 1, and 10 mg/L, for Cbz doses of 50 and 100 µmol/L, and for Flx doses of 5 and 10 µmol/L. Tube content (with viable and dead cells) was stained with Annexin V-FITC (AnnV) and propidium iodide (PI) using the flow cytometry Annexin V Kit (Bio Rad, Hercules, California, USA) to count live (AnnV−, PI−), early apoptotic (AnnV+, PI−), apoptotic (AnnV+, PI+), and dead cells (AnnV−, PI+) on a Cytoflex SRT sorter using its software (Beckman Coulter Life Sciences, Indianapolis, IN, USA).

Results are reported as the percentage of live, early apoptotic, late apoptotic, or dead cells compared to negative controls obtained from three independent experiments done in triplicate.

For further experiments we used only Cbz, Flx, and PNSP doses that left more than 90 % of cells viable to avoid bias that may arise from dead, unviable, or damaged cells.

### Measurement of oestrogen receptor activity

Oestrogen receptor activity in T47D-KBluc cells was determined for the test substances or their mixtures using the luciferase assay (Promega, Madison, WI, USA, Cat. Nos.: E1500 and E1501). The assay is based on the binding to oestrogen-responsive elements by oestrogen receptors that make part of the T47D-KBluc cell DNA sequence. Binding to these elements induces gene transcription that produces luciferase, which converts beetle luciferin (assay reagent) to a luminescent product oxyluciferin. The intensity of its luminescence is proportional to oestrogen receptor activation.

Our measurements followed the OECD test No. 455 (5). The responsiveness of the test system was checked using diethylstilbestrol (DES) as positive control and fulvestrant as negative control. The quality control of the assay confirmed ≥four-fold mean luciferase activity compared to negative/vehicle control on each plate and no interference by the test substances (PSNPs and neurodrugs) with the assay components and readouts.

The assay was run on T47D-KBluc cells that were first cultured for a week in a medium in which 10 % FBS was replaced by 10 % charcoal-stripped FBS to attenuate interferences from serum hormones. After one week, the cells were seeded in white opaque flat-bottom Nunc™ MicroWellTM 96-well microplates (Thermo Fisher Scientific, Waltham, MA, USA) at a density of 2×10^4^ cells per well, each containing 100 µL of culture medium containing 5 % (v/v) charcoal-stripped FBS and kept there at 37 °C and 5 % CO_2_ for 24 h to attach to the wells. Followed a 48-hour treatment with different concentrations of PSNPs, Cbz, and Flx alone or their mixtures. Untreated cells were used as negative control and cells treated with 10 nmol/L DES as positive control. After the treatment, oestrogen receptor activity was measured on a SpectraMax iD3 microplate reader (Molecular Devices, San Jose, CA, USA) using the Promega luciferase assay kit as described above.

Results are expressed either as the percentage of fold luminescent signal inductions compared to negative or positive controls.

### Statistical analysis

Statistical analysis was run on GraphPad Prism6 (GraphPad Software, San Diego, CA, USA). Statistical significance was determined by one-way ANOVA followed by Dunnett’s multiple comparison test with negative control values and set to P<0.05.

## RESULTS AND DISCUSSION

### Physico-chemical characteristics and stability of PSNPs

**Figure 1:**
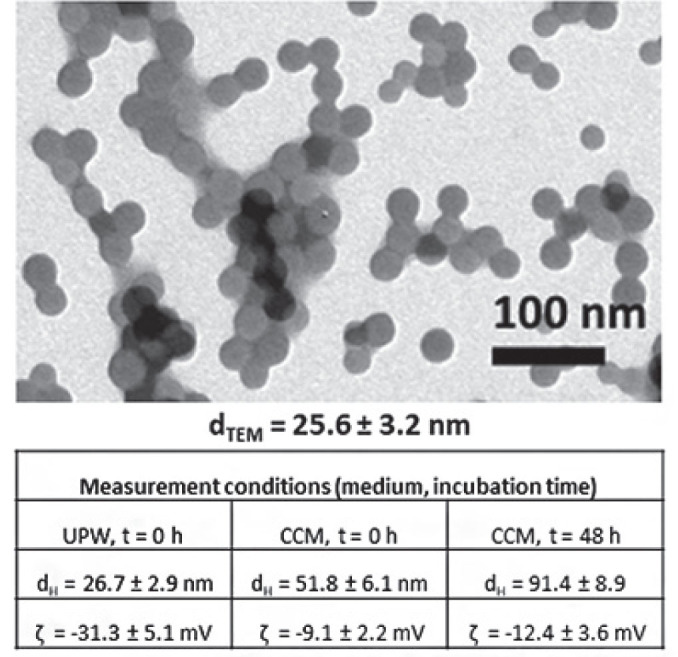
Transmission electron micrograph (TEM) and physico-chemical properties of 10 mg/L polystyrene nanoparticles (PSNPs) in ultrapure water (UPW) and the RPMI-1640 cell culture medium (CCM) at baseline (t=0 h) and after 48 h (t=48 h) at 25 °C. *d*_TEM_ – PSNP dispersion in ultrapure-water, *d*_H_ – PSNP hydrodynamic diameter in UPW and CCM; ζ – PSNP zeta potential in in UPW and CCM

Figure 1 shows the shape, size, size distribution, and zeta potential of the PSNPs used in this study. They were of spherical shape and primary diameter (*d*_TEM_) of 25.6±3.2 nm, confirming the producer’s declaration (Phosphorex). Their hydrodynamic diameter (*d*_H_) was greater than *d*_TEM_ due to hydration and protein corona shell formation on the nanosurface, while further increase in *d*_H_ after 48 h indicates slight aggregation due to increased ionic strength of the cell culture medium (RPMI-1640). The zeta potential was negative and also increased after 48 h.

### Cytotoxicity of PSNPs, Cbz, Flx, and their mixtures

**Figure 2:**
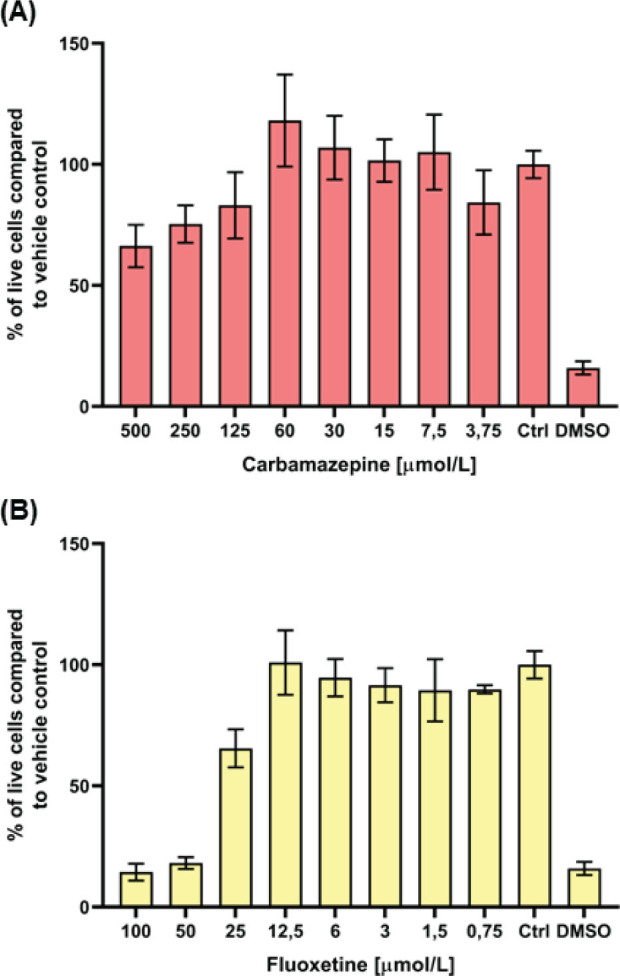
Viability of T47D-KBluc cells treated with A) carbamazepine (Cbz) and B) fluoxetine (Flx) established with the MTS assay. Untreated cells were used as negative control (Ctrl) and cells treated with 10 % (v/v) DMSO as positive control (DMSO). Results are given as the percentage of Ctrl and calculated as mean values from three independent experiments. Standard deviations are given as error bars, and values that are significantly different from Ctrl are marked with * (P<0.05)

The MTS assay showed that doses below 60 µmol/L for Cbz and below 12.5 µmol/L for Flx did not affect the viability of T47DKBluc cells (Figure 2), while the IC_50_ values were 174.8 and 30.17 µmol/L, respectively.

**Figure 3:**
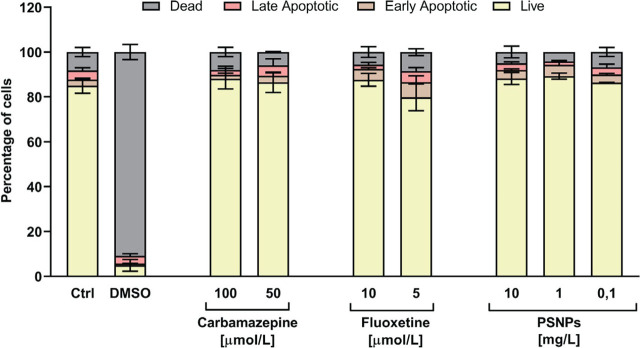
Viability and apoptosis in T47D-KBluc cells treated with PSNPs, Cbz and Flx established with flow cytometry. Untreated cells were used as negative control (Ctrl) and cells treated with 10 % (v/v) DMSO as positive control (DMSO). Results are given as the percentage of live, early apoptotic, late apoptotic, and dead cells compared to Ctrl and calculated as mean values from three independent experiments. Standard deviations are given as error bars

Further flow cytometry showed that none of tested substances at given doses (0.1, 1, 5, and 10 mg/L for PSNPs; 1, 5, 10, 50, and 100 µmol/L for Cbz; and 0.1, 0.5, 1, 5, and 10 µmol/L for Flx) induced significant damage to the T47D-KBluc cells (Figure 3), so we proceeded with these doses in further luciferase assay experiments.

### Oestrogen receptor activity in cells exposed to PSNPs, Cbz, Flx, and their mixtures

**Figure 4:**
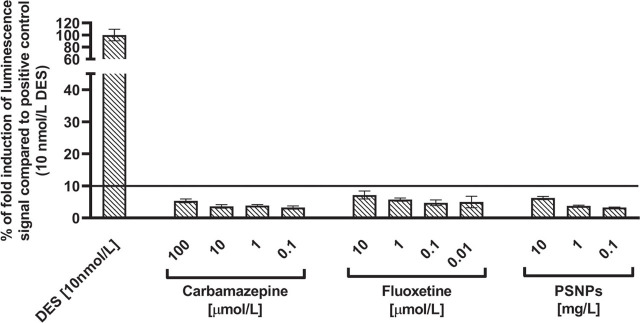
Oestrogen receptor activity in response to carbamazepine (Cbz), fluoxetine (Flx), and polystyrene nanoparticle (PSNP) treatment of T47D-KBluc cells. Results are shown as the percentage of fold induction of luminescent signal in comparison with positive control (10 nmol/L diethylstilbestrol, DES) and represent mean values from three independent experiments done in triplicate. Standard deviations (SD) are given as error bars. Values above the black line denote endocrine disrupting response according to the OECD test No. 455 (5)

Endocrine disrupting effects of Cbz and Flx in mixtures with PSNPs were determined by comparing cell oestrogen receptor activity in respective mixtures (Cbz-PSNPs and Flx-PSNPs) with that of individual components (Cbz, Flx, or PSNPs). As described in the OECD test No. 455, a substance can be considered endocrine disruptor if oestrogen receptor response is equal to or exceeds 10 % of the response obtained with 10 nmol/L of DES. In view of these guidelines, the tested substances alone did not induce oestrogen receptor activity (Figure 4).

**Figure 5:**
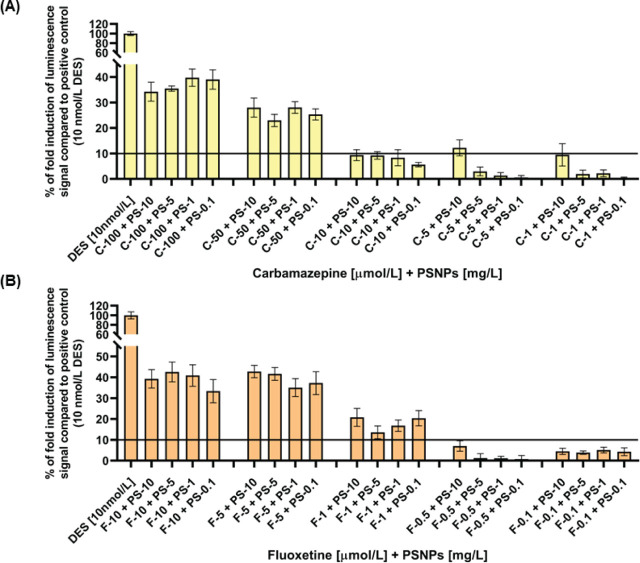
Oestrogen receptor activity in response to PSNP mixtures with (A) carbamazepine (Cbz) and (B) fluoxetine (Flx) in T47D-KBluc cells. Results are shown as the percentage of fold induction of luminescent signal in comparison with positive control (10 nmol/L diethylstilbestrol, DES) and represent mean values from three independent experiments done in triplicate. Standard deviations (SD) are given as error bars. Values above the black line denote endocrine disrupting response according to the OECD test No. 455 (5)

However, Cbz-PSNP and Flx-PSNP mixtures show quite alarming results in terms of health hazard and evidence that nanoplastics do behave like a Trojan horse in T47D-KBluc cells regardless of their concentration (Figure 5). Cbz and Flx mixed with PSNPs evoked strong endocrine disrupting response (≥10 % of oestrogen response to 10 nmol/L DES) starting with the doses of 50 and 1 µmol/L, respectively.

Interestingly, oestrogen receptor response did not depend on PSNP concentrations. This may be owed to greater PSNP aggregation in the cell culture medium when particle concentrations are higher, which may decrease specific surface area available for interaction with cells and drug absorption. Furthermore, the observed response did not show dose dependence for the two higher drug doses (50 and 100 µmol/L of Cbz, 5 and 10 µmol/L of Flx), most likely because of nanosurface saturation.

## CONCLUSION

This *in vitro* study presents the first evidence of endocrine disrupting activity of complex mixtures containing polystyrene nanoparticles and neuroactive drugs. Our findings have confirmed our initial hypothesis that substances combined with nanoparticles will induce endocrine disruptive response at doses which do not provoke such response when applied alone. This calls for the revision of current health hazard assessment practices for environmental pollutants to include mixtures of plastic nanoparticles and pharmaceuticals in toxicity evaluation, considering that oestrogen receptor activation can lead to the development of various cancers mediated by various key events, such as increased proliferation and migration of cells, oxidative stress, non-genomic signalling and inflammatory response as evidenced by the AOP-Wiki, an open web platform launched by the OECD to support hazard assessment (35,36,37).
